# Genre Painting and the World's Kitchen

**DOI:** 10.3201/eid1101.AC1101

**Published:** 2005-01

**Authors:** Polyxeni Potter

**Affiliations:** Centers for Disease Control and Prevention, Atlanta, Georgia, USA

**Figure:**
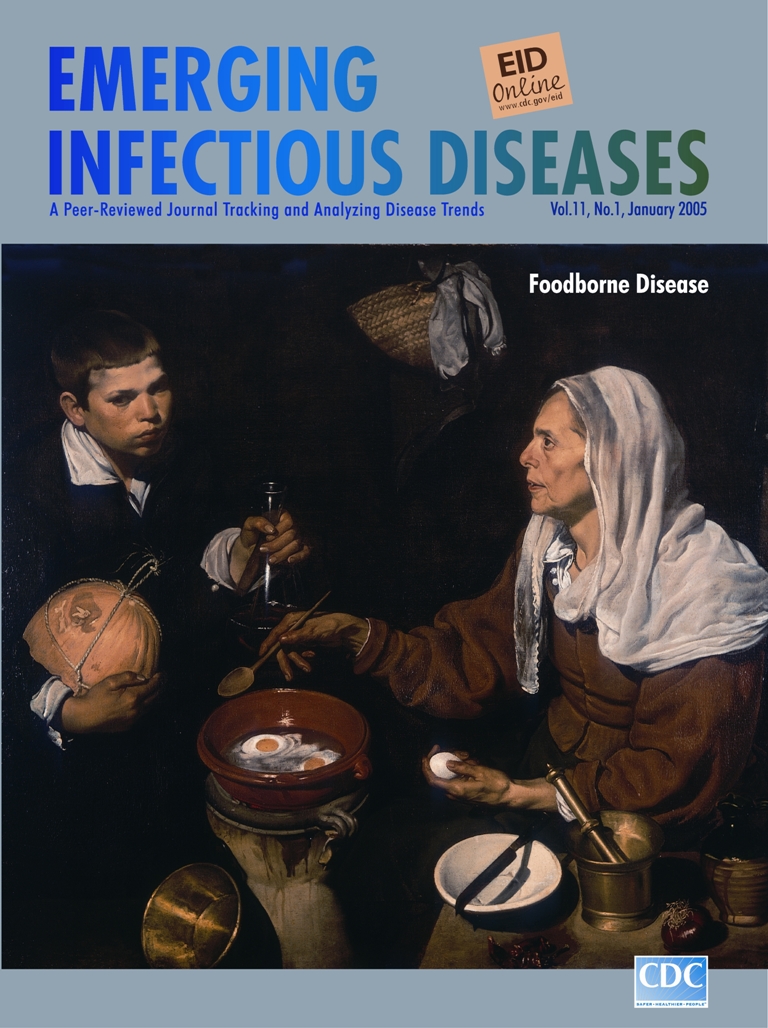
**Diego Velázquez (1599–1660). An Old Woman Cooking Eggs (1618)** Oil on canvas (100.5 cm x 119.5 cm). The National Gallery of Scotland, Edinburgh

"Tell me what you eat, I will tell you who you are," boasted famed gastronome Jean Anthelme Brillat-Savarin (1755–1826) (*1*). A man of many interests, among them archaeology, astronomy, and chemistry, Savarin wrote treatises on economics and history, but his fascination with food was what most informed and entertained readers and followers in his native France and around the world. In nature and on the table, quite apart from its direct link to human survival, food has been an object of intrigue featured prominently in art throughout history. From ancient times and particularly during the development of genre painting in the Middle Ages and later, food—its appearance, abundance, or decay—has been a popular subject.

In 17th-century Spain, genre painting (scenes of everyday life) reached new heights with the work of Diego Velázquez. In a style reminiscent of Caravaggio, Velázquez created and popularized a new genre, the kitchen or tavern scene (bodegón), which showed peasants eating or preparing meals and the objects they used to assemble and serve them. These objects (still life), prominently displayed in realistic terms including their imperfections, assumed a life of their own, introducing a new naturalism in Spanish painting, which had been dominated by the ideal beauty of classical and academic themes (*2*).

Velázquez grew up in the cosmopolitan climate of Seville, southern Spain, along the banks of the Guadalquivir, an area also home to Cervantes, Lope de Vega, and other luminaries of the Spanish Golden Age. Like the great literature of that era, his art concerned itself with the life, culture, and traditions of the people. He apprenticed with influential biographer, theoretician, and artist Francisco Pacheco, who later wrote about his student: "After five years of education and training, I married him to my daughter, moved by his virtue, integrity, and good parts and by the expectations of his disposition and great talent" (*3*).

Soon a member of the Seville painters' guild, Velázquez moved from bodegón to portraits and was summoned to the court, where he received his first commission to paint King Philip IV, a discerning patron of the arts. He was appointed court painter, a position of great privilege, which gave him access to royal collections including paintings by the Venetian Renaissance master Titian, who greatly influenced the development of his style. The artist led a quiet life, interrupted only by his travels to Italy, sponsored by the king. During his first journey, he traveled with Flemish Baroque master Peter Paul Rubens, who was also influential in his artistic career.

"To go to Madrid to see the Velázquez" was Monet's wish near the end of his life (*4*). This wish, expressed in a letter to a friend, reflects the mystique associated with Velázquez' work and the breadth of its influence on all modern art schools, even if limited to 100 or so surviving works. His painting showed exceptional mastery of space and light and exuded naturalness and restraint, both in its objectivity and choice of colors, often browns and ochers. Velázquez was gifted with exacting technique and preferred to paint from life. In spite of his meticulous depiction of reality, he seemed more interested in the tensions between reality and appearance than in reality itself (*5*).

Velásquez painted An Old Woman Cooking Eggs (on this month's cover of Emerging Infectious Diseases) when he was 19 years old. In this kitchen scene, the common utensils used in preparing food (mortar and pestle, pots, ladles, bowl, jugs) have at least as important a place as the preparers themselves. Provocatively in the foreground and along the edges of the painting, these objects seem to contain in their clay, wood, glass, brass, copper, pewter, or other essence the light that defines them against the dark background. The eggshell, the straw of the basket, the skin of the melon and onion, the texture of linen and string, showcase the artist's virtuoso performance in capturing their likeness.

The food preparers, transfixed by some unknown concern, seem removed and distant from the food and from each other. They go through the motions of cooking, but their minds are elsewhere. The boy, cradling a trussed melon, leans forward with a glass cruet containing oil, wine, or some other liquid. The old woman tending the food is staring intently ahead, otherwise preoccupied. On a ceramic heating plate, the pan is tipped forward to show the eggs in various stages of congealing. The curved shadow of the knife over the bowl, the moist surface of the pan above the egg whites, the gleaming copper pot against the shadows of the room confirm the artist's interest in the integrity and dignity of these objects and the people who use them, even if he does not indulge us with their concerns underneath the surface.

These concerns, apart from the underlying threat of decay through the relentless passage of time, a common theme in still-life painting, would be many, even if they were only limited to food. The 17th-century Spanish diet was known for its parsimony. A main concern in the common kitchen was the long-term availability of food. The safety of food, a more modern concern, was probably not on the mind of Velásquez' food preparers. Unlike our contemporary equivalents, they would have known little about the dangers surrounding food. Nor would they have understood Savarin, whose sensitive 18th-century palate might have recoiled at the sight of eggs poaching slowly in oil on a clay stove.

An ancient staple, eggs have run the gamut from plentiful protein to gourmet delicacy. Yet, basic food and epicurean aspirations converge at one point: safety. With high levels of *Salmonella* Enteriditis in shell eggs (*6*), adequate cooking and proper temperature of the eggs overrule tradition, challenging the consistency of the sauce and the moment of delivery to the table. In our times, safety issues concerning not only eggs but all foods beg a different interpretation of another well-known Savarin aphorism, "The destiny of a nation depends on the manner in which it feeds itself."
